# Free‐energy calculations of residue mutations in a tripeptide using various methods to overcome inefficient sampling

**DOI:** 10.1002/jcc.24488

**Published:** 2016-09-16

**Authors:** Michael M. H. Graf, Manuela Maurer, Chris Oostenbrink

**Affiliations:** ^1^ Institute of Molecular Modeling and Simulation, Department of Material Sciences and Process Engineering, BOKU, University of Natural Resources and Life SciencesMuthgasse 18AT‐1190ViennaAustria

**Keywords:** free‐energy calculations, one‐step perturbation, replica exchange, thermodynamic integration, GROMOS

## Abstract

Previous free‐energy calculations have shown that the seemingly simple transformation of the tripeptide KXK to KGK in water holds some unobvious challenges concerning the convergence of the forward and backward thermodynamic integration processes (i.e., hysteresis). In the current study, the central residue X was either alanine, serine, glutamic acid, lysine, phenylalanine, or tyrosine. Interestingly, the transformation from alanine to glycine yielded the highest hysteresis in relation to the extent of the chemical change of the side chain. The reason for that could be attributed to poor sampling of φ_2_/ψ_2_ dihedral angles along the transformation. Altering the nature of alanine's C_β_ atom drastically improved the sampling and at the same time led to the identification of high energy barriers as cause for it. Consequently, simple strategies to overcome these barriers are to increase simulation time (computationally expensive) or to use enhanced sampling techniques such as Hamiltonian replica exchange molecular dynamics and one‐step perturbation. © 2016 The Authors. Journal of Computational Chemistry Published by Wiley Periodicals, Inc.

## Introduction

This study roots in previous investigations on the oligopeptide binding Protein A (OppA), one of the most abundant periplasmic proteins in gram‐negative bacteria such as *Escherichia coli* and *Salmonella typhimurium*.^1^ OppA binds nutrients (i.e., peptide fragments) in the periplasm and shuttles them to a transmembrane transporter, thereby playing a key role in nutrient transport.^2^ The accepted peptide fragments can have two to five residues with no preference for their composition, which confers OppA a broad substrate promiscuity.^3^, ^4^, ^5^ However, because of the negative charge at its binding site, OppA has a preference toward positively charged substrates, particularly lysine containing tripeptides.^6^ Experimental studies revealed that the substrate promiscuity is especially pronounced for the central amino acid of KXK tripeptides, where X may represent 20 natural and 8 non‐natural amino acids.^7^, ^8^, ^9^ The 28 tripeptides bind with a wide range of binding affinities, which could, however, not straightforwardly be correlated to the nature (polar, apolar, aromatic, or charged) of the central residue.^10^

A recent study employing molecular dynamics simulations focuses on the tripeptides KGK, KAK, and KSK because of their structural similarity but significantly different experimental binding free energies (Δ*G*_bind,exp_) to OppA.^11^ To compute the differences in relative binding free energies (Δ*G*_bind,sim_) of these tripeptides to OppA, MD simulations of transformations from one tripeptide to the other were conducted within the protein environment and freely in water. For the transformations of KAK → KGK and KGK → KAK in water, a relatively large discrepancy in Δ*G*_mut_ between the forward and backward process was reported (hysteresis). This observation was surprising because the central residues only differ in a single methyl group. Differences in the φ_2_/ψ_2_ dihedral angle distributions in the forward and backward processes at corresponding *λ*‐values were suggested as a reason for the observed hysteresis. Consequently, an aim of the current study was to rationalize the hysteresis of this particular transformation and to compare it to KXK → KGK transformations of a systematically selected set of central residues, with X being serine (small, polar), glutamic acid and lysine (large, charged), or phenylalanine and tyrosine (large, aromatic).

Central residue transformations in the KXK tripeptide of the aforementioned amino acids, but notably not alanine to KGK, have recently been investigated by Bieler and Hünenberger,^12^ however, with an eye on their newly developed *λ*‐LEUS approach.^13^ The *λ*‐LEUS approach was developed to overcome sampling shortcomings, for example, in orthogonal φ_2_/ψ_2_ dihedral angle distributions of the KXK → KGK transformations during thermodynamic integration (TI). It combines *λ*‐dynamics^14^ with local elevation^15^ and umbrella sampling^16^, ^17^ and hence, is rather complex to implement as a standardly used simulation protocol. The free‐energy changes of the tripeptide transformations calculated according to *λ*‐LEUS or TI showed significant differences in mean absolute deviations with respect to the average free energy difference (*n* = 10) between different transformations but also for single transformations calculated according to both methods.^12^ In the current study, we revisit the previously investigated KXK → KGK tripeptide transformations in water.^11^, ^12^ On the one hand, we integrate all KXK → KGK tripeptide transformations in water tested so far^11^, ^12^ in the present study. The most interesting transformation, that is, the one with the highest hysteresis in relation to the conducted alchemical transformation, will then be selected to identify the reason for discrepancies in the forward and backward processes. In this study, we explicitly focus on fairly straightforward approaches, which are readily available, and therefore we refrain from developing new methodologies or using elaborate sampling schemes such as *λ*‐LEUS.

## Methods

### Simulation settings and free energy calculations

The coordinates for the initial tripeptide structures were prepared with the MOE software package.^18^ All MD simulations were performed with the GROMOS11 software package^19^ with the 45A3 force field^20^ to stay close to the previously published studies.^11^, ^12^ All systems were energy minimized *in vacuo* using the steepest‐descent algorithm before placing them into a periodic, pre‐equilibrated, rectangular box of SPC water.^21^ The minimum solute‐to‐wall and maximum solute‐to‐solvent distances were set to 0.8 and 0.23 nm, respectively. Energy minimization of the solvent using the steepest‐descent algorithm was performed while positionally restraining the solute to relax unfavorable atom–atom contacts between solvent and solute. To equilibrate all systems, initial velocities were randomly assigned according to a Maxwell–Boltzmann distribution at 60 K and the systems were propagated for 20 ps. Rototranslational constraints were used for all solute atoms.^22^ In each of the three subsequent 20 ps MD simulations, the temperature was increased by 60 K. In a last 1 ns equilibration step, the temperature was set to the final temperature of 298 K.

During production runs, the pressure (1 atm) and temperature (298 K) were kept constant using the weak‐coupling scheme^23^ with coupling times of 0.5 and 0.1 ps, respectively. The isothermal compressibility was set to 4.575 × 10^−4^ kJ^−1^ mol nm^3^, and two separate temperature baths were used for the solute and solvent. The SHAKE algorithm was used to constrain bond lengths,^24^ allowing for a 2 fs time‐step. Nonbonded interactions were calculated using a triple range scheme: Interactions within a short‐range cutoff of 0.8 nm were calculated at every time step from a pair list that was generated every fifth step. At every fifth time‐step, interactions between 0.8 and 1.4 nm were also calculated explicitly and kept constant between updates. To account for a homogenous medium outside the long‐range cutoff, a reaction‐field contribution was added to the electrostatic interactions and forces,^25^ with a relative dielectric constant of 61 as appropriate for the SPC water model.^26^

### Thermodynamic integration

In TI,^27^ two Hamiltonians describing states A and B are alchemically connected over a path defined by a scaling parameter *λ*. At *λ* = 0, the Hamiltonian, H(0), describes state A, and at *λ* = 1 state B. At intermediate *λ* values, H(λ) is continuous between A and B and represents—possibly unphysical—intermediates.^28^ The free energy difference on a path along state A to B can be obtained by numerically integrating the curve ${〈\partial H/\partial \lambda 〉}_{\lambda }$ as a function of *λ* according to following equation:^27^

(1) $$\Delta G_{A\to B}=G_{B}-G_{A}=\int_{0}^{1}{〈\frac{\partial H}{\partial \lambda }〉}_{\lambda }d\lambda$$

where the angular brackets denote the ensemble average of the derivative of H(λ) with respect to *λ*, obtained from independent simulations at discrete intermediate values of *λ*. The Hamiltonian is parametrized with respect to *λ* to include a softcore potential avoiding singularities in the derivatives.^29^ This is governed by two softcore parameters, *α*_VdW_ and *α*_CRF_, for Lennard‐Jones and electrostatic interactions, respectively. To monitor convergence of the TI simulations, the free‐energy difference between the forward (state A to B, *λ* increasing from 0 to 1) and the backward (state B to A, *λ* decreasing from 1 back to 0) process was compared, which is denoted hysteresis. The hysteresis should approach zero for converged simulations.

For one alchemical transformation, at least 11 equidistant *λ* values were used. Additional *λ* points were introduced on abruptly changing ${〈\partial H/\partial \lambda 〉}_{\lambda }$ values to achieve a smooth and more accurate mutation curve for the TI. After an equilibration time of 50 ps, production simulations of 1 ns per *λ* value were routinely conducted for all transformations. The simulations for one mutation were run sequentially, that is, the initial configuration at a certain *λ* value was taken from the final configuration of the equilibration simulation at the previous *λ* value. The first configuration used for the backward mutation was obtained from the last snapshot of the forward production simulation at *λ* = 1. The coordinates and energies were stored every 0.5 ps and 0.1 ps, respectively. Statistical error estimates are obtained from block averaging and extrapolation to infinite block length.^30^

High energy barriers that are difficult to cross during MD simulations caused surprisingly high hysteresis for the initial TI of the KAK → KGK transformation. To overcome these barriers, different approaches were used (see the results section for details):

1. Extending the production runs to 10 ns per *λ* value;
2. Overcoming barriers by (A) Increasing the softness parameter *α*_VdW_ from 0.5 to 1.0 nm^2^ to lower repulsive energies from overlapping atoms^11^, ^29^; (B) Excluding specific intramolecular interactions, with coordinate trajectories written out every 0.1 ps for post‐MD analysis; (C) Changing the mass and/or size of the alanine side chain to change its momentum;
3. Mixing conformations during Hamiltonian replica exchange simulations (see below);
4. Using a single unphysical reference state and the one‐step perturbation (OSP) (see below).

### Hamiltonian replica exchange molecular dynamics

Hamiltonian replica exchange molecular dynamics (HREMD) can efficiently be used for TI and is routinely used to enhance sampling by running multiple parallel, independent MD simulations of the same system.^31^, ^32^ The replicas all have slightly different Hamiltonians H which are defined by their *λ*‐values and allowed to exchange according to the Metropolis criterion.^33^, ^34^, ^35^ With this approach, conformational space can more readily be sampled if high‐energy barriers only exist in certain states.^28^, ^35^, ^36^, ^37^ After the simulations are finished an ensemble average at each *λ*‐value is generated by sorting the trajectory of each replica according to *λ*. In the end, the free energy difference between the replicas can be computed according to eq. (1).

In the current study, 11 parallel MD simulations were performed starting at 11 equidistant *λ* values for 1 ns each. Neighboring replicas were allowed to exchange their *λ* values every 2 ps. Coordinates and energies were stored every 0.5 ps and 0.1 ps, respectively.

### One‐step perturbation

The free energy difference between related states A and B can also be computed from MD simulations by the one‐step perturbation (OSP) approach.^38^, ^39^ OSP is an application of the free energy perturbation technique developed by Zwanzig^40^ and makes use of an (unphysical) reference state *R*, of which the Hamiltonian overlaps with that of states A and B. Using the perturbation formula

(2) $$\Delta G_{R\to A}=G_{A}-G_{R}=-k_{B}T\ln{〈e^{-\left(H_{A}-H_{R}\right)/k_{B}T}〉}_{R}$$

the free energy of transforming *R* to one of the end states (Δ*G_R_*_→__*A*_ or Δ*G_R_*_→__*B*_) can be computed, where *k*_B_ is the Boltzmann constant, *T* the temperature, and $H_{A}$ and $H_{R}$ the Hamiltonians of the real compound A or the reference state, respectively. ${〈\cdot 〉}_{R}$ represents the ensemble average over all configurations generated during a simulation using the Hamiltonian of *R*. The free energy difference between the end‐states (Δ*G_A_*_→__*B*_) can be calculated as the difference of Δ*G_R_*_→__*B*_ and Δ*G_R_*_→__*A*_.

To guarantee sufficient overlap of the reference state *R* within the tripeptide K*R*K with alanine and glycine for the KAK → KGK transformation during OSP, the C_β_ of *R* was treated as a neutral softcore atom with Van der Waals parameters of a CH_3_ group, and a softness parameter *α*_VdW_ of 1.51. As the C_β_ in Ala is neutral in this (united atom) force field, no softcore parameter for the electrostatic interactions was needed. The total simulation time was 10 ns, coordinates and energies were stored every 0.1 ps.

## Results and Discussion

As described in the introduction, residue mutations of the tripeptide KXK → KGK were already investigated in previous studies employing either TI or a combination of *λ*‐dynamics and local‐elevation umbrella‐sampling (*λ*‐LEUS).^11^, ^12^ Especially for the KAK → KGK transformation, a significant hysteresis between the forward and backward process was observed and attributed to poor orthogonal sampling of φ_2_/ψ_2_ dihedral angle distributions.^11^ The sampling problem was tackled with the *λ*‐LEUS approach,^12^ in which the coupling parameter *λ* is treated as a dynamic variable, which is subsequently biased to cycle repeatedly between states A and B, with a prolonged residence time at the end‐states. The cyclic sampling of *λ* allows the physical system to overcome barriers as long as these disappear at some value of *λ*. In *λ*‐LEUS, the bias on the sampling of *λ* is build up and maintained using local elevation, followed by an umbrella sampling phase. This approach proved to be successful but is rather complex to implement as a standard simulation procedure. Moreover, the reason for the poorly sampled φ_2_/ψ_2_ dihedral angle distributions, ultimately leading to the observed hysteresis, was not thoroughly investigated in those previous studies. In the present work, these issues are tackled with a set of consecutive approaches, on the basis of which the results and discussion section is divided. First, we identify the minimal test system with TI calculations to investigate the sampling problem and subsequently, we attempt to tackle that issue by (1) longer sampling per *λ* point during TI, (2) reducing barriers during TI, (3) mixing conformations during Hamiltonian replica exchange molecular dynamics simulations (HREMD), or (4) using a single unphysical reference state in OSP.

### Minimal test system

**Figure 1:**
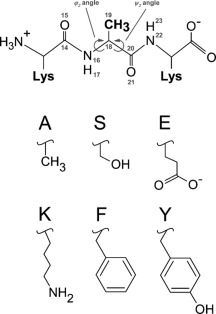
In the upper part, the tripeptide KAK, atom numbering scheme around the central alanine, and the φ_2_/ψ_2_ dihedral angles are indicated. The side chains of the central residue X in tripeptide KXK investigated in this study are shown below. X can be one of the following amino acids: alanine (A), serine (S), glutamic acid (E), lysine (K), phenylalanine (F), or tyrosine (Y). The KXK → KGK mutations were accomplished by transforming the atoms that are present in X but not in G into dummy atoms and by modifying the C_*α*_ atom from a CH to a CH_2_ group. Note that all aliphatic H atoms are implicit in the applied united atom force field.

**Figure 2:**
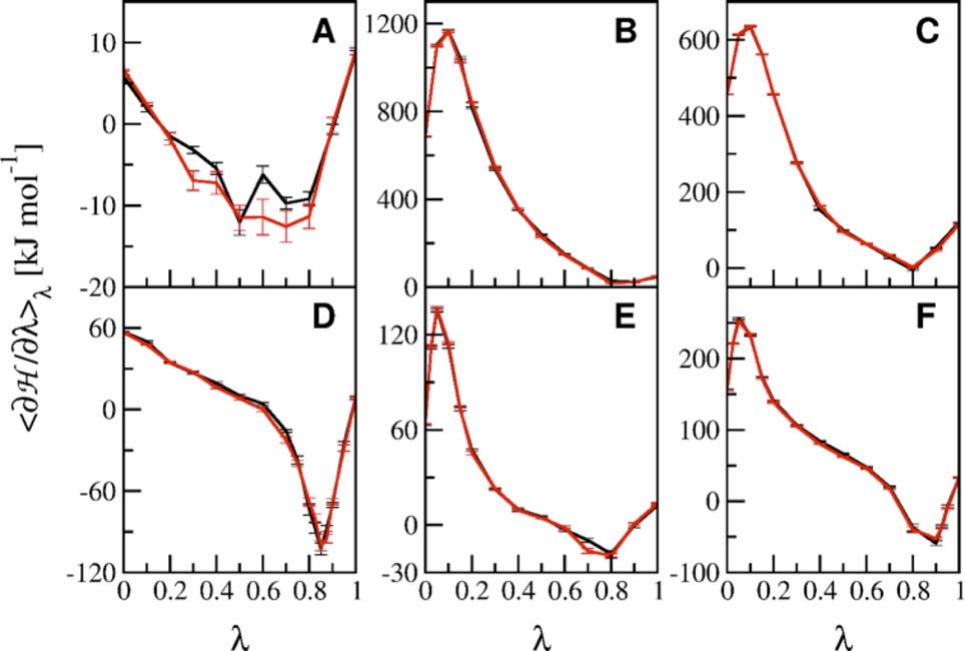
Values of ${〈\partial H/\partial \lambda 〉}_{\lambda }$ as a function of *λ* for the TI processes between KXK (*λ* = 0) and KGK (*λ* = 1) in solution, whereas X corresponds to following amino acids: alanine (panel A), glutamic acid (panel B), lysine (panel C), phenylalanine (panel D), serine (panel E), and tyrosine (panel F). The forward (black) and backward (red) perturbations were all conducted with *α*_VdW_ = 0.5 and *α*_CRF_ = 0.5 nm^2^ with 1 ns per *λ*. [Color figure can be viewed at wileyonlinelibrary.com]

TI calculations of KXK → KGK mutations were conducted, where the central amino acid X was alanine, serine, glutamic acid, lysine, phenylalanine, or tyrosine (Fig. 1). The TI calculations were performed for 1 ns per *λ* point and *α*_VdW_ = 0.5 and *α*_CRF_ = 0.5 nm^2^ for both, the forward and backward process, with 11 *λ* points equidistantly distributed for 0 ≤ *λ* ≤ 1. Significant deviations between TI profiles were observed at high curvature regions previously, which could be counteracted by introducing additional *λ* points.^11^ Consequently, the same strategy was employed for regions with increased curvature in the present study to obtain smooth TI curves (e.g., in Fig. 2 around *λ* = 0.1 in panels B, C, E, and F or between *λ* = 0.8 and 0.9 in panel D).

The hysteresis, that is, the difference between integrated values of the forward and backward TI curves, ranges between 0.6 and 1.6 kJ mol^−1^ (Table 1). Although the hysteresis for the KAK → KGK transformation is amongst the highest of the investigated transformations it has the lowest associated statistical error. Because the total hysteresis may still be camouflaged by cancellation of errors, we also computed the integrated absolute hysteresis between backward and forward processes over the entire curve in the last column of Table 1. The integrated absolute hysteresis for all tested systems ranges between 1.6 and 8.1 kJ mol^−1^ and is actually the second lowest for the KAK → KGK transformation (note that the scale in Fig. 2A is significantly different from the other panels).

**Table 1:** Free energy differences (Δ*G*) in kJ mol^−1^ for the forward and backward transformation of solvated KGK and KXK.

	Δ*G* (kJ mol^−1^)			
Transformation	Forward	Backward	Hysteresis	Integrated absolute hysteresis
KAK → KGK	−3.9 ± 0.7	−5.3 ± 0.5	1.4 ± 0.9	1.8 ± 0.9
KEK → KGK	388.1 ± 9.3	387.3 ± 8.7	0.9 ± 12.7	8.1 ± 12.7
KFK → KGK	0.9 ± 1.0	−0.6 ± 0.9	1.5 ± 1.3	2.8 ± 1.3
KKK → KGK	209.3 ± 5.5	209.9 ± 5.1	0.6 ± 7.5	3.9 ± 7.5
KSK → KGK	22.8 ± 1.6	22.2 ± 1.7	0.6 ± 2.3	1.6 ± 2.3
KYK → KGK	72.7 ± 3.6	71.1 ± 2.9	1.6 ± 4.6	2.6 ± 4.6

The values were calculated from the TI curves depicted in Figure 2, which are based on simulations with *α*_VdW_ = 0.5, *α*_CRF_ = 0.5 nm^2^, and 1 ns per *λ*.

**Figure 3:**
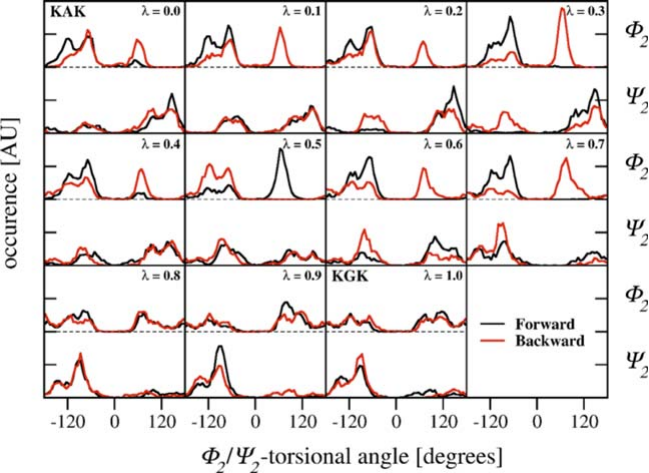
Distributions for the φ_2_‐ and ψ_2_‐angles of the tripeptide in the forward (black) and backward (red) TI process between KAK and KGK (corresponding to panel A in Figure 2A or the first line in Tables 1 and 2), calculated for all *λ* values from simulations with *α*_VdW_ = 0.5 with 1 ns per *λ*. [Color figure can be viewed at wileyonlinelibrary.com]

Although serine has a slightly bigger side chain than alanine, the hysteresis for the KSK → KGK transformation is only 0.6 kJ mol^−1^ and the integrated absolute hysteresis 1.6 kJ mol^−1^, which are both the lowest values of all investigated processes. A comparison of the φ_2_/ψ_2_ dihedral angle distributions at corresponding *λ* values in the forward and backward transformations for the KAK → KGK (Fig. 3) and KSK → KGK (Fig. S1 Supporting Information) transformation explains that observation. For KAK → KGK, the φ_2_/ψ_2_ dihedral angle distributions at specific *λ* values do not match, reflected in the nonoverlapping black and red curves in a single panel. This indicates poor sampling of phase space because of the inability to cross barriers of that system. Although these issues are present at some *λ* values during the KSK → KGK transformation as well (Fig. S1 Supporting Information), the curves for the forward and backward process do match for the majority of φ_2_/ψ_2_ dihedral angle distributions at the appropriate *λ* values. Consequently, the difference in φ_2_/ψ_2_ dihedral angle distributions at corresponding *λ* values in the forward and backward transformations was identified as the origin of the larger hysteresis for the KAK → KGK transformation, as observed earlier.^11^

This observation is striking from a chemical point of view because the transformation from an alanine to a glycine is the smallest change among all tested systems but still yields the third highest hysteresis in our test set. In relation to the number of atoms that change in the central residue, it yields by far the highest hysteresis among all investigated transformations. While for the larger side chains, the hysteresis may be due to inefficient solvent reorganization and solvation processes, the KAK → KGK transformation truly is the smallest system and reduces the problem to barriers in the main chain. To investigate the differing forward and backward φ_2_/ψ_2_ dihedral angle distributions during this transformation in more detail, we chose this as suitable test system for further simulations.

### Longer sampling time per *λ* point during TI

**Figure 4:**
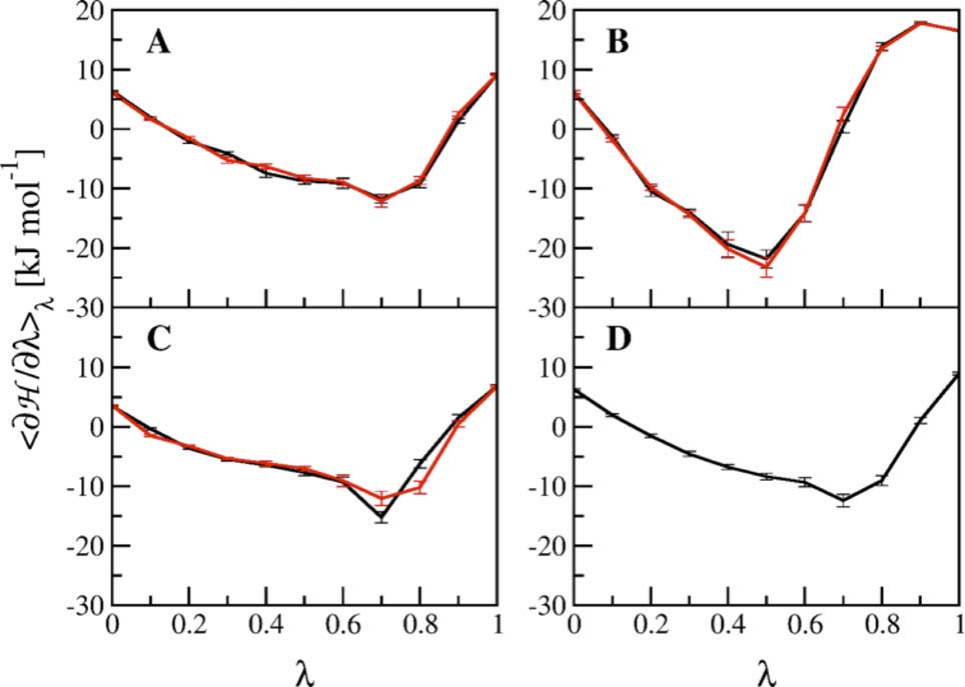
Values of <∂ H/∂*λ*>_*λ*_ as a function of *λ* for the transformations of KAK to KGK in solution. Panels A to C show the TI processes between KAK (*λ* = 0) and KGK (*λ* = 1) for the forward (black) and backward (red) perturbations. The TI processes were conducted similarly as for Figure 2, with following changes: in panel A, the simulation time was 10 ns per *λ*; in panel B, *α*_VdW_ was 1.0; in panel C, a slightly modified Hamiltonian was utilized, excluding specific intramolecular interactions and the coordinates were written out every 50th step for subsequent calculations. In panel D, HREMD was used. [Color figure can be viewed at wileyonlinelibrary.com]

**Figure 5:**
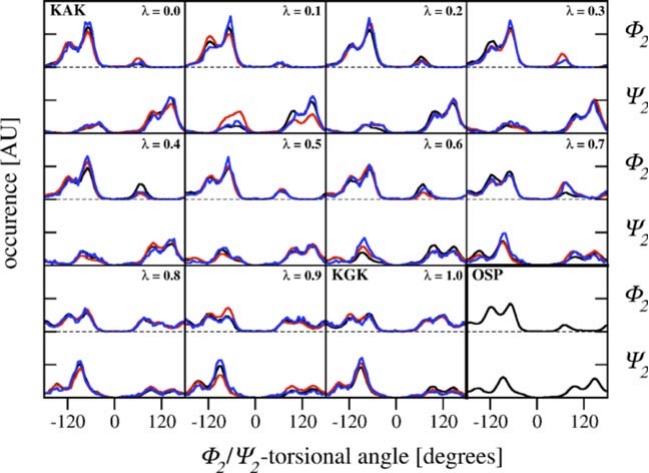
Distributions for the φ_2_‐ and ψ_2_‐angles of the tripeptide in the forward (black) and backward (red) TI process between KAK and KGK, calculated for all *λ* values from simulations with *α*_VdW_ = 0.5 and 10 ns per *λ* (corresponding to the second line in Table 2). The φ_2_‐ and ψ_2_‐angles distributions derived from HREMD are depicted in blue (compare to the last line in Table 2 and panel D in Fig. 4). The distributions for the 10 ns OSP simulations of the reference state in water are shown in the lower right panel (compare to Table 3). [Color figure can be viewed at wileyonlinelibrary.com]

To minimize the hysteresis in the KAK → KGK transformation, our first approach was to prolong the simulations from 1 to 10 ns per *λ* point. The free energy differences between the forward and backward processes, the corresponding hysteresis, and the integrated absolute hysteresis are depicted in the first and second line in Table 2. The results clearly show that the hysteresis and the integrated absolute hysteresis as well as their statistical errors are significantly reduced for TI with 10 ns per *λ* point. A comparison of ${〈\partial H/\partial \lambda 〉}_{\lambda }$ as a function of *λ* between panels A in Figures 2 and 4 corroborates the better fit of the forward and backward transformations in the prolonged simulations. Moreover, the φ_2_/ψ_2_ dihedral angle distributions at corresponding *λ* values are essentially the same in the forward and backward transformations for the TI with 10 ns per *λ* point (black and red line in Fig. 5). To ensure that the sampling of the φ_2_/ψ_2_ dihedral angles are the main reason of the hysteresis, we have also computed the radial distributions of the distance between the lysine sidechain atoms and the C_β_ of the central residue (Fig. S6 Supporting Information). The general shape of these distributions is very similar in the forward and backward simulations already after 1 ns, while the distributions become virtually the same after 10 ns.

**Table 2:** Free energy differences (Δ*G*) in kJ mol^−1^ calculated from TI or HREMD, respectively.

			Δ*G* (kJ mol^−1^)			
Method	Total simulation time (ns)	*α*	Forward	Backward	Hysteresis	Integrated absolute hysteresis
TI	22	0.5	−3.9 ± 0.7	−5.3 ± 0.5	1.4 ± 0.9	1.8 ± 0.9
TI	220	0.5	−4.1 ± 0.4	−3.9 ± 0.4	0.2 ± 0.6	0.6 ± 0.6
TI	22	1.0	−3.8 ± 0.6	−3.8 ± 0.6	0.1 ± 0.8	0.6 ± 0.8
TI	220	1.0	−3.9 ± 0.6	−4.2 ± 0.7	0.3 ± 0.9	0.6 ± 0.9
TI^a^	22	0.5	−5.0 ± 0.8	−4.8 ± 0.7	0.2 ± 1.1	2.2 ± 1.1
TI^b^	22	0.5	−2.7 ± 0.7	−2.3 ± 0.6	0.4 ± 0.9	2.3 ± 0.9
TI^c^	22	0.5	−4.6 ± 0.6	−5.0 ± 0.6	0.4 ± 0.8	1.7 ± 0.8
HREMD	11	0.5	−4.1 ± 0.4			

For TI, the forward and backward transformation of solvated KAK and KGK was computed.

a Slightly modified Hamiltonian, excluding specific intramolecular interactions; additionally, the coordinates were written out every 50th step for subsequent calculations.

b Alteration of the side chain in the alanine topology from —CH_3_ to an increased mass (corresponding to a —CH_2_—OH group).

c Alteration of the side chain in the alanine topology from —CH_3_ to an artificial —CH_2_ group (mass and atom type).

Although longer sampling per *λ* point resolves the issue of poor hysteresis, this comes at a high computational cost as the total simulation time increases tenfold from 22 ns to 220 ns. Consequently, we tried other approaches to solve the sampling issue and possibly find an explanation for the large hysteresis in the TI with 1 ns per *λ* point.

### Reduced barriers during TI

Insufficient sampling of the φ_2_/ψ_2_ dihedral angle distributions was identified as the reason for the comparably large hysteresis in the KAK → KGK transformation, caused by energy barriers that are difficult to cross during the simulations. We tried several approaches to overcome these barriers, reach convergence faster, and ultimately lower the hysteresis: (A) Increasing the softness parameter *α*_VdW_ from 0.5 to 1.0. (B) Excluding specific intramolecular interactions. (C) Increase or decrease the mass of the alanine side chain to allow for higher or lower momentum, respectively. Alternatively, one could reduce the barriers by reducing the force constant of the torsional‐angle potential‐energy term. However, in the currently used parameter set, the barriers due to these potential‐energy terms amount to 1 kJ/mol only and the main contribution is due to the nonbonded interactions.

#### Increased Softness Parameters

During an alchemical transformation, the derivatives of the Hamiltonian with respect to *λ* may become infinitely large as surrounding atoms occupy the positions of non‐interacting dummy particles. To avoid this, the softcore potential is used.^29^ As a result, interactions for disappearing atoms may be weakened already at intermediate states, hence reducing barriers between different conformations. The softcore parameters are therefore important when transforming noninteracting dummy atoms into their fully interacting counterpart in the course of a TI. For the end states, which are described by the pure Hamiltonians of compound A (*λ* = 0) and B (*λ* = 1), the softcore potential is irrelevant. Although the curvature for the intermediate *λ* points might differ, the integrated free‐energy differences of the whole TI process remains unaffected. The correlation between softcore potential and calculation efficiency were the subject of previous studies.^11^, ^41^, ^42^ In the present work, we increased the softness parameter *α*_VdW_ from 0.5 to 1.0.

Figure 4, panel B shows the values of ${〈\partial H/\partial \lambda 〉}_{\lambda }$ as a function of *λ* for the TI with 1 ns per *λ* point and *α*_VdW_ of 1.0. As mentioned previously, the increased softness affects the curvature for 0 < *λ* < 1, which is indeed elevated compared to the TI with *α*_VdW_ of 0.5 (Fig. 4, panel A). The free energy differences for the KAK → KGK transformation are given in the third line in Table 2 and are within the statistical uncertainty of the values obtained from the 10 ns simulations with *α*_VdW_ = 0.5. The hysteresis is 1.3 kJ mol^−1^ lower compared to the TI with a softness of 0.5 and 1 ns per *λ* point. Moreover, the integrated absolute hysteresis as well as the associated error estimates are significantly smaller in the TI with a softness of 1.0. The φ_2_/ψ_2_ dihedral angles at the corresponding *λ* values show a more similar distribution between the forward and backward process compared to the TI with a softness of 0.5 (compare Figs. 3 and S2 Supporting Information). Not surprisingly, prolongation of the TI with increased softness to 10 ns per *λ* point did not lead to further improvement in hysteresis or integrated absolute hysteresis as was the case for the TI with a softness of 0.5, indicating that the simulations are converged (Table 2). Additionally, the hysteresis and the integrated absolute hysteresis from the TI with a softness of 1.0 and 1 or 10 ns per *λ* point all fall within the statistical error estimates.

Increasing the softness parameters indeed enables the alanine side chain to more easily cross high energy barriers and to more readily adopt the wider sampling that is required for the KGK end state in the course of the alchemical change. As the softness of the —CH_3_ group in alanine seemed to play a large role, we next addressed the nonbonded interactions of this atom more explicitly.

#### Exclusion of Intramolecular Interactions

Arguably, the reduced hysteresis due to an increased softness parameter was rather fortuitous. To come to a more generally applicable solution to achieve low hysteresis for the KAK → KGK transformation, the tripeptide's Hamiltonian was slightly modified throughout the process. In detail, the nonbonded interactions for alanine's C_β_ atom with its 1,4‐neighbors C_14_, H_17_, O_21_, and N_22_ (refer to Fig. 1 for atom numbering) were excluded from the nonbonded energy calculations in addition to the regularly excluded 1,3‐neighbors. In the subsequent TI with 1 ns per *λ* point and a softness of 0.5, this led to a TI profile, which was close to that of the reference TI with 10 ns per *λ* point and a softness of 0.5 (compare panels A and C in Fig. 4). The smoother TI profile can again be attributed to very well matching φ_2_/ψ_2_ dihedral angle distributions at corresponding *λ* values in the forward and backward transformations (Fig. S3 Supporting Information).

However, the smooth profile for ${〈\partial H/\partial \lambda 〉}_{\lambda }$ in panel C of Figure 4 was obtained by an incorrect representation of the system's Hamiltonian. To correct for this, the entire free energy profile was corrected by considering the addition of the exclusions as a potential energy bias and unbiasing the estimate of ${〈\partial H/\partial \lambda 〉}_{\lambda }$ like in the umbrella sampling approach. The reweighted ensemble average for the correct Hamiltonian without the additional exclusions was obtained from

(3) $${〈\frac{\partial H}{\partial \lambda }〉}_{\text{NoExcl}}=\frac{{〈\frac{\partial H_{\text{NoExcl}}}{\partial \lambda }e^{-\left(H_{\text{NoExcl}}-H_{\text{Excl}}\right)/k_{B}T}〉}_{\text{Excl}}}{{〈e^{-\left(H_{\text{NoExcl}}-H_{\text{Excl}}\right)/k_{B}T}〉}_{\text{Excl}}}$$

where the subscript Excl and NoExcl refer to Hamiltonians and sampling appropriate for the situation with additional exclusions and without these, respectively. After this correction, the hysteresis between the forward and backward transformations is 1.2 kJ mol^−1^ lower compared to the initial TI with a softness of 0.5 and 1 ns per *λ* point (compare first and fifth line in Table 2). However, its associated statistical error is highest and the integrated absolute hysteresis second highest of all tested TI protocols (Table 2). The free energy differences for both processes are 0.9 kJ mol^−1^ lower compared to the reference TI with a softness of 0.5 and 10 ns per *λ* point. Not surprisingly, the reweighting using eq. (3) increases the uncertainty in the ensemble averages.

Next, we intended to investigate in more detail the difference between the KAK → KGK and KSK → KGK transformations, as the latter one seemed to suffer less from hysteresis than the former one.

#### Changing the Mass and Character of the Alanine Side Chain

##### Mass of a —CH_2_—OH Group

In the KSK → KGK transformation, the φ_2_/ψ_2_ dihedral angles were mostly equally distributed for the forward and backward process at corresponding *λ* values, which is in contrast to the transformation starting with KAK. To rationalize why the structurally similar alanine behaves so differently from serine, we tested if increasing the mass of the alanine side chain —CH_3_ (15.0350 u) to that of serine —CH_2_—OH (31.0344 u) would lead to increased momenta allowing the crossing of high energy barriers in a TI with 1 ns per *λ* point and a softness of 0.5. This would potentially allow for better sampling of phase space and therefore more equally distributed φ_2_/ψ_2_ dihedral angles in the forward and backward processes, ultimately leading to lower hysteresis. Indeed, this approach decreased the hysteresis significantly by 1.0 kJ mol^−1^, but increased the integrated absolute hysteresis by 0.5 kJ mol^−1^ compared to the initial TI with 1 ns per *λ* point and a softness of 0.5 (compare first and sixth line in Table 2; refer to Fig. S4 Supporting Information for the φ_2_/ψ_2_ dihedral angle distributions). The free energy difference for the forward and backward processes are 1.4 and 1.6 kJ mol^−1^ higher compared to the reference TI with 10 ns per *λ* point and a softness of 0.5. Note that in GROMOS, the free energy difference due to changes in the kinetic energy are included in the full Hamiltonian, because, due to the use of SHAKE, the momenta and positions are no longer strictly uncoupled. The changes in the free energy are readily explained from this contribution.

##### Mass and Size of a —CH_2_ Group

In a next step we investigated if the improved hysteresis for the serine sidechain may simply be caused by the slightly smaller size of the united atom —CH_2_ group, as compared to the —CH_3_ group. Therefore, we changed the GROMOS integer atom code and mass to that of a —CH_2_ group to 13 and 14.0270 u, respectively. As was the case for the heavier alanine side chain, the TI with 1 ns per *λ* point and a softness of 0.5 resulted in a hysteresis that was lowered by 1.0 kJ mol^−1^. The integrated absolute hysteresis decreased only slightly by 0.1 kJ mol^−1^ compared to the initial TI with 1 ns per *λ* point and a softness of 0.5 (compare first and seventh line in Table 2). The much lower hysteresis can again be attributed to more uniformly distributed φ_2_/ψ_2_ dihedral angles especially in the alanine state (Fig. S5 Supporting Information) compared to the TI with a regular alanine (Fig. 3). The free energy difference for the forward and backward processes are 0.5 and 1.1 kJ mol^−1^ lower compared to the reference TI with 10 ns per *λ* point and a softness of 0.5 as we have physically changed one of the end states.

Overall, the various modifications to the TI setups and system Hamiltonians suggest that the barriers leading to insufficient sampling of the φ_2_/ψ_2_ dihedral angles only need to be reduced slightly to improve the sampling. In a next step, instead of changing the softness parameters, the exclusions, or the mass and atom type, we tried to enhance sampling and therefore lower the hysteresis by exchanging the coordinates of replicas from different simulations with slightly different simulation parameters in HREMD.

### Mix conformations during HREMD

To retrieve more uniform φ_2_/ψ_2_ dihedral angle distributions at corresponding *λ* values, we additionally used HREMD.^34^ This method can be used to improve sampling of alchemical free energy calculations in TI, where each parallel replica represents a state along the reaction coordinate *λ*.^35^ The free energy difference derived from HREMD lies within the statistical error estimates for the forward and backward processes of the reference TI and the ${〈\partial H/\partial \lambda 〉}_{\lambda }$ profile is very similar to the one of the reference TI (compare panels A and D in Fig. 4). This agreement can be attributed to φ_2_/ψ_2_ dihedral angles that are distributed similarly as for the forward and backward process of the reference TI (compare the blue line to the black and red lines in Fig. 5). Thanks to the regular switches between *λ* values, any replicate can reversibly move to states along the transformation (here the KGK state) at which the barriers are minimal and different conformations are sampled. Subsequent switches back to the state with increased barriers mixes in the new conformations into the conformational ensemble with the appropriate weight. The reversible visiting of different states along the transformation is exactly the key aspect of the *λ*‐LEUS aproach that was described earlier.^12^ There, a single simulation is biased to repeatedly visit all *λ* values while in the HREMD approach this is naturally enforced for all individual replicas. Consequently, the HREMD approach yields very accurate results at very small computational costs of only 11 ns total simulation time. Moreover, with current simulation codes, this represents a simple simulation setup.

### Using a single unphysical reference state in OSP

To complement the TI and HREMD results, OSP calculations^38^ were performed for K*R*K → KAK and K*R*K → KGK transformations, where *R* depicts an unphysical, softcore reference state. The Hamiltonian of the reference state *R* is constructed such that one single simulation trajectory of *R* is sufficient to sample relevant conformations of A and G. With this procedure, it is possible to calculate the free energy difference (Δ*G*) for the transformation of *R* to each individual end state using eq. (2) (middle column in Table 3). The Δ*G* difference (ΔΔ*G*) for both transformations is −3.9 ± 0.3 kJ mol^−1^ (last column in Table 3), which corresponds to the KAK → KGK transformation. This value is in excellent agreement with the reference TI (−4.1 ± 0.4 kJ mol^−1^ forward; −3.9 ± 0.4 kJ mol^−1^ backward) and HREMD (−4.1 ± 0.4 kJ mol^−1^), depicted in the second and last lines in Table 2. The overlap in sampling can here be visualized by the distribution of the φ_2_/ψ_2_ dihedral angles that are characteristic for both, the alanine and glycine end‐states (black line in the lower right corner in Fig. 5). Except for the sampling of the φ_2_ dihedral angle at +120°, the reference state shows a distribution that has features of both KAK and KGK. With a total simulation time of only 10 ns, the OSP approach is most efficient in terms of calculation time compared to all other tested methods. However, it is obvious that this method is not readily applicable for any transformation, but will be limited to small, neutral amino acid side chains.^43^

**Table 3:** Free energy differences (Δ*G*) calculated from OSP in kJ mol^−1^ between the solvated reference state (K*R*K) and KAK or KGK, respectively.

	kJ mol^−1^	
Transformation	Δ*G*	ΔΔ*G*_KAK→KGK_
K*R*K → KAK	6.5 ± 0.3	−3.9 ± 0.3
K*R*K → KGK	2.6 ± 0.2	

To guarantee sufficient overlap with the real compounds KAK and KGK, the C_β_ of *R* was treated as a neutral soft atom with Van der Waals parameters of a CH_3_ group and *α*_VdW_ = 1.51. The total simulation time was 10 ns.

## Conclusions

In the tripeptide transformation KXK → KGK with the central residue being alanine, serine, glutamic acid, lysine, phenylalanine, or tyrosine, hysteresis between the forward and backward transformation has been observed previously.^11^, ^12^ The hysteresis could be linked to poor sampling of the φ_2_/ψ_2_ dihedral angles. The sampling problem was either tackled with *λ*‐LEUS,^12^ which is rather difficult to implement as a standard simulation procedure, or by simply increasing the softness parameter *α*_VdW_ from 0.5 to 1.0.^11^ Here, we have investigated the reason for the sampling problem of the orthogonal φ_2_/ψ_2_ dihedral angle degrees of freedom in the KXK tripeptide in more detail and have suggested simple and efficient ways to overcome the sampling problem.

We identified the KAK → KGK tripeptide transformation as the most suitable test system during TI because of its relatively large hysteresis compared to the conducted chemical change of the central residue. We confirmed poor sampling of the φ_2_/ψ_2_ dihedral angles as the main cause for hysteresis. Poor sampling in turn could be attributed to too high energy barriers during the transformation. Several different approaches were tested to overcome these high energy barriers: (1) increasing simulation time from 1 to 10 ns per *λ* point; (2A) increasing the softness parameter *α*_VdW_ from 0.5 to 1.0, modifying excluded atoms (2B), the mass, or the size (2C) of the alanine C_β_ atom. These approaches were complemented by (3) HREMD, and (4) OSP simulations, which were found to be most efficient in terms of calculation time. An alternative, which was not studied in the current work, would be to restrain the conformational freedom of the backbone dihedrals during the alchemical change and subsequently compute the free energies of releasing these restraints in the endstates. This would likely reduce the hysteresis in the alchemical step, but shift the sampling issue to the releasing of the restraints in the most flexible molecule (i.e., KGK).

Increasing the simulation time resulted in very low hysteresis, at the cost of a tenfold increase in simulation time. Changing the softness parameter *α*_VdW_ from 0.5 to 1.0 resulted in the lowest hysteresis and lowest integrated absolute hysteresis of all considered approaches. An elevated or lowered mass for alanine's C_β_ both resulted in comparable (low) hystereses, however, the integrated absolute hysteresis was in the range of the initial TI with 1 ns per *λ* point and a softness of 0.5. Excluding 1,4‐neighbors from the nonbonded interactions of alanine's C_β_ had a similar effect. The lowered hysteresis could be attributed to more uniformly distributed φ_2_/ψ_2_ dihedral angles in the forward and backward process. Because changing the mass and therefore the momentum as well as excluding 1,4‐neighbors from the nonbonded interactions of alanine's C_β_ all led to significantly better sampling of φ_2_/ψ_2_ dihedral angles, we conclude that slightly too high energy barriers indeed exist for the KAK → KGK transformation. Easily crossing these barriers is only possible with adjusted side chain parameters, which is remarkable, given the fact that the transformation from alanine to glycine represents a very small chemical change. HREMD and OSP for the KAK → KGK transformation both yielded similar Δ*G* values at the lowest computational cost (11 and 10 ns of total simulation time, respectively). Moreover, the obtained free energy differences are in very good agreement with the reference TI with 10 ns per *λ* point and a softness of 0.5.

Overall, the reason for poor sampling of φ_2_/ψ_2_ dihedral angle distributions was determined to come from subtle barriers in the energy landscape, which ultimately leads to hysteresis in the KAK → KGK tripeptide transformation. Simple changes in the simulation protocol (increased softness, use of HREMD) seem to be sufficient to significantly improve the consistency of the results. The current work may be seen as a simple example where standard protocols do not work straightforwardly, but slight modifications by an expert user are needed to obtain reliable and reproducible results.

## Supporting information

Supporting Information
