# Supplementary material for: Free‐energy calculations of residue mutations in a tripeptide using various methods to overcome inefficient sampling
Source: J Comput Chem. 2016 Sep 16;37(29):2597–605. doi: 10.1002/jcc.24488 (PMC5082540; doi:10.1002/jcc.24488)

Supplementary Material

Free-energy calculations of residue mutations in a tripeptide using various methods to overcome inefficient sampling

Michael M.H. Graf,[1] Manuela Maurer,[1] and Chris Oostenbrink[1]

[1] *Michael M.H. Graf, Manuela Maurer, Chris Oostenbrink*

Institute of Molecular Modeling and Simulation, BOKU, University of Natural Resources and Life Sciences, Muthgasse 18, AT-1190 Vienna, Austria

Correspondence to: Prof. Chris Oostenbrink (E-mail: *chris.oostenbrink@boku.ac.at*)

Contents

Figures S1-S5: additional φ_2/ψ_2 distributions for various simulations.

Figure S6: radial distributions of lysine sidechain atoms relative to the C_β of the central residue.

Figure S1

Distributions for the φ_2- and ψ_2-angles of serine in the forward (black) and backward (red) TI process between KSK and KGK, calculated for all λ values from simulations with $\alpha_{VdW} = 0.5$, $\alpha_{CRF} = 0.5\ nm^2$, and 1 ns per λ (corresponding to the fifth line in Table 1 and panel E in Figure 2).

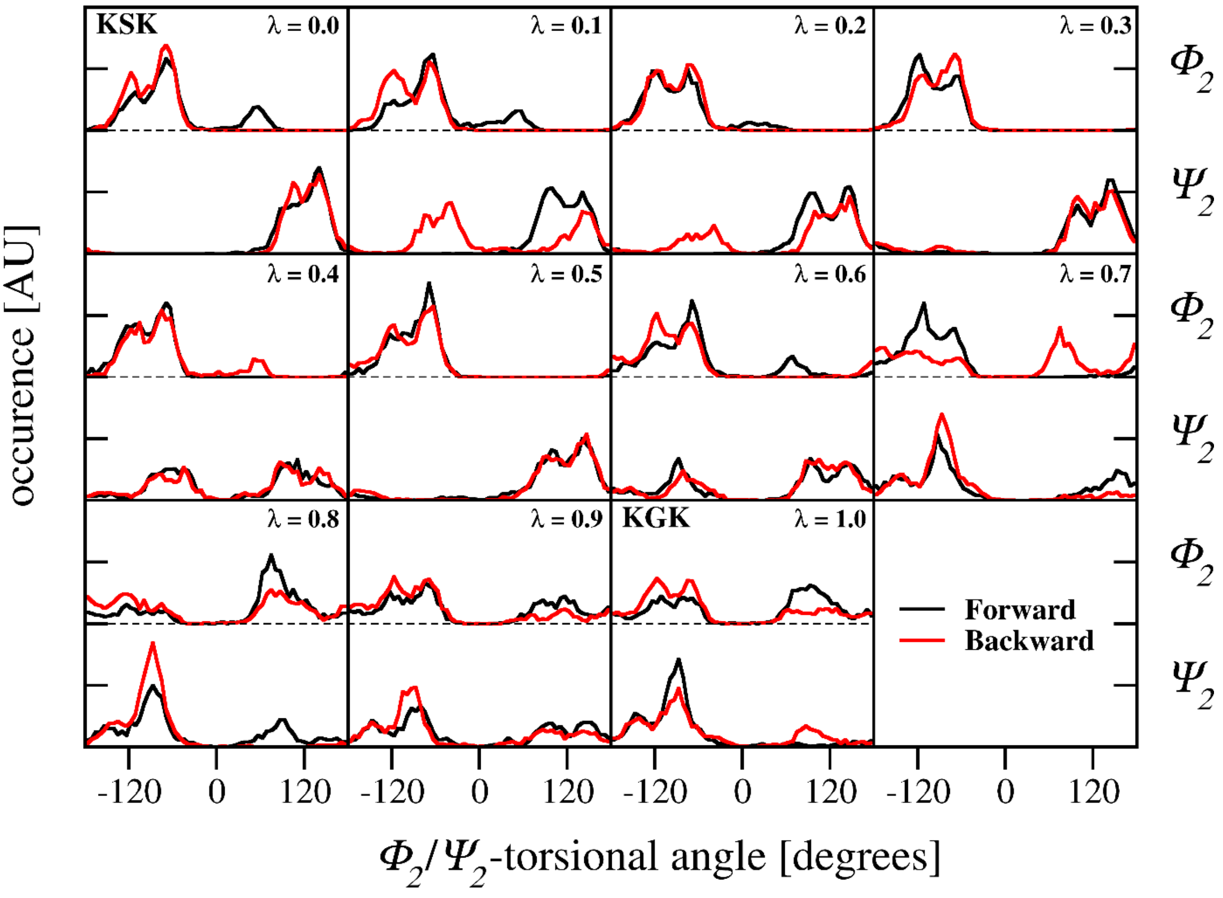

Figure S2

Distributions for the φ_2- and ψ_2-angles of alanine in the forward (black) and backward (red) TI process between KAK and KGK, calculated for all λ values from simulations with α_{VdW} = 1.0 and 1 ns per λ (corresponding to the third line in Table 2 and panel B in Figure 4).

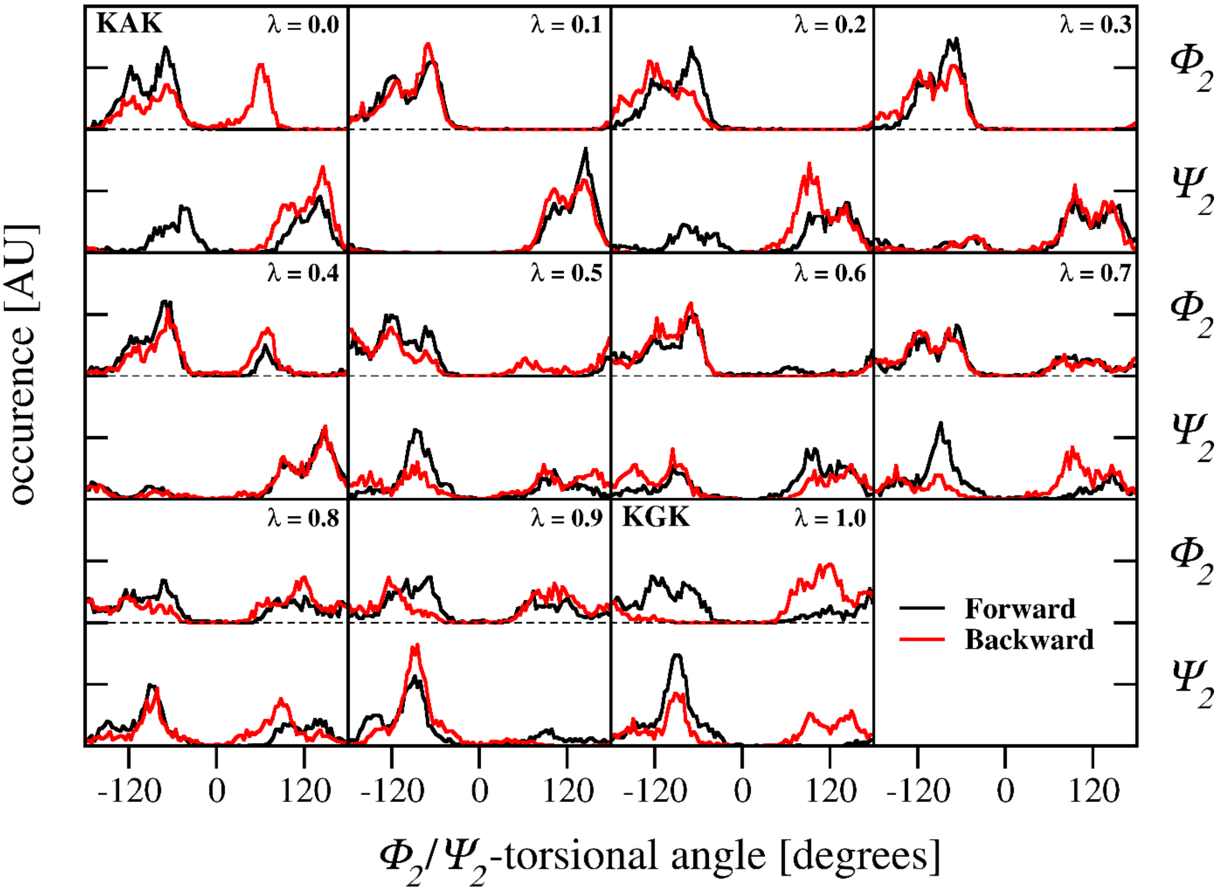

Figure S3

Distributions for the φ_2- and ψ_2-angles of alanine in the forward (black) and backward (red) TI process between KAK and KGK, calculated for all λ values from simulations with α_{VdW} = 0.5, 1 ns per λ, and a slightly modified Hamiltonian, excluding specific intra-molecular interactions. The coordinates were written out every 50th step for subsequent use of Equation 3. Compare to the fifth line in Table 2 and panel C in Figure 4).

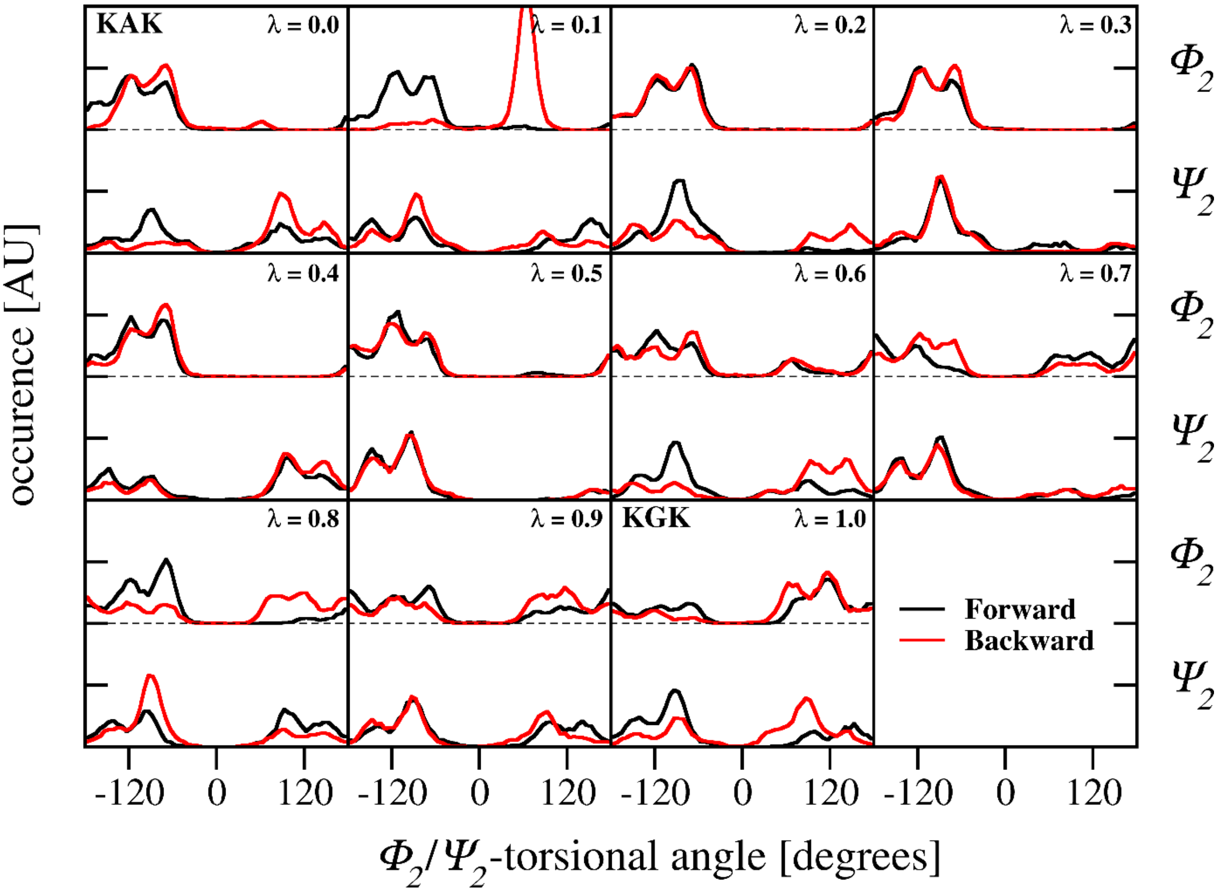

Figure S4

Distributions for the φ_2- and ψ_2-angles of alanine in the forward (black) and backward (red) TI process between KAK and KGK, calculated for all λ values from simulations with α_{VdW} = 0.5 and 1 ns per λ (corresponding to the sixth line in Table 2). For this TI for this TI, the mass of alanine's C_β was increased to 31.0344 u to reflect that of a serine $-CH_2-OH$.

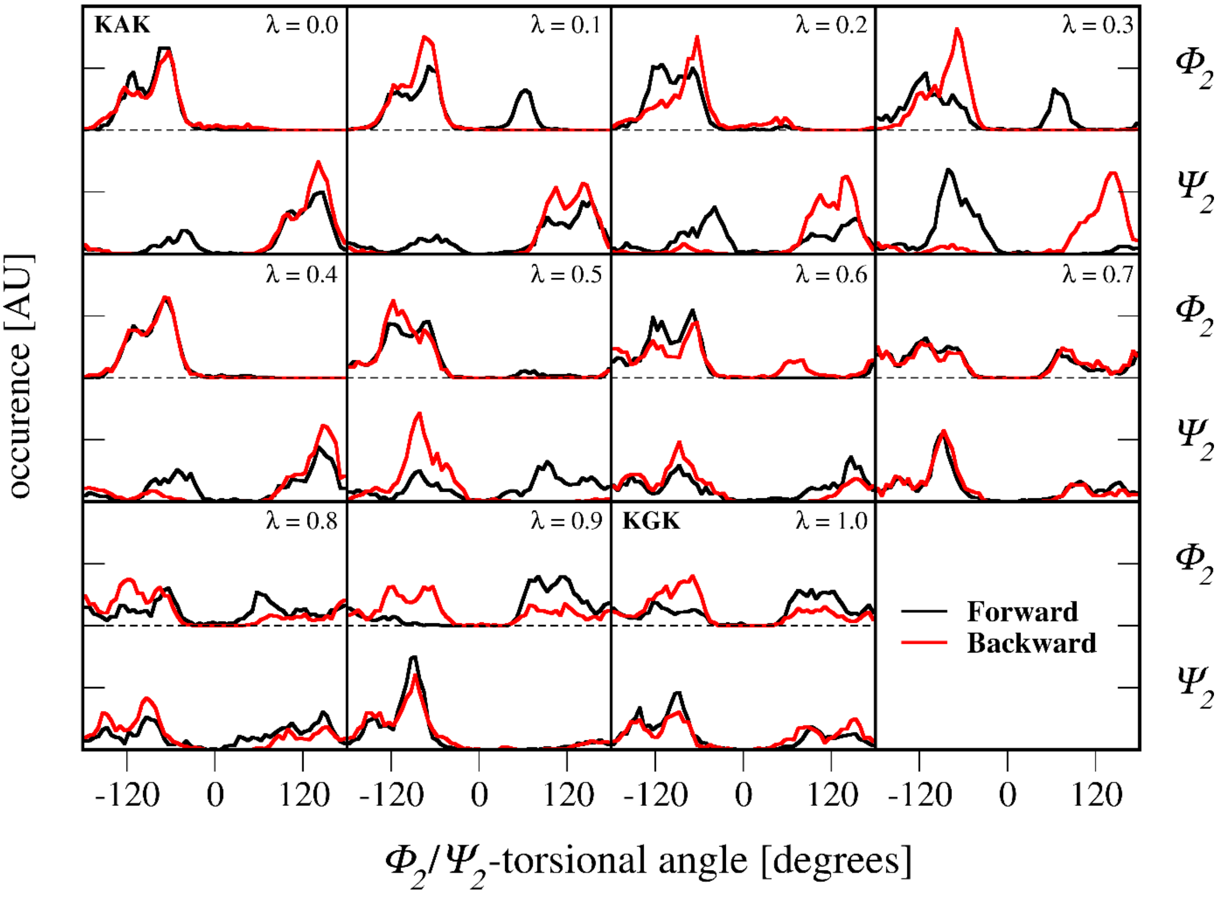

Figure S5

Distributions for the φ_2- and ψ_2-angles of alanine in the forward (black) and backward (red) TI process between KAK and KGK, calculated for all λ values from simulations with α_{VdW} = 0.5 and 1 ns per λ (corresponding to the seventh line in Table 2). In the topology for this TI, following changes were made to alanine's C_β in order to reflect a $-CH_2$: Its mass was decreased to 14.0270 u and its GROMOS integer atom code was changed to 13.

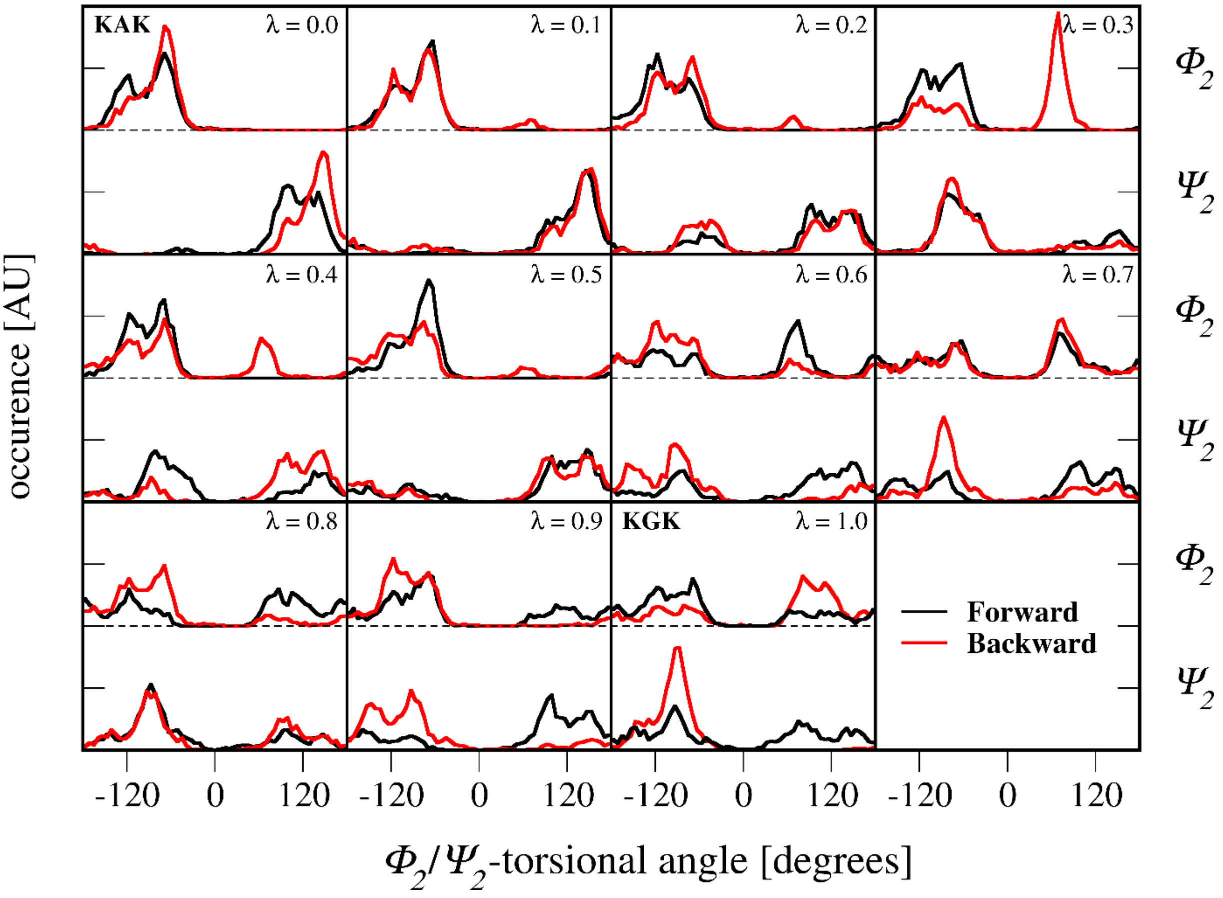

Figure S6

Radial distribution functions of the lysine sidechain atoms surrounding the central C_β in alanine (left panel) and the corresponding dummy atom in glycine (right panel). The distributions are computed from the endstates of the forward (black curves) and backward (red curves) TI process between KAK and KGK, averaged over 1 ns (dashed curves) or 10 ns (solid curves).

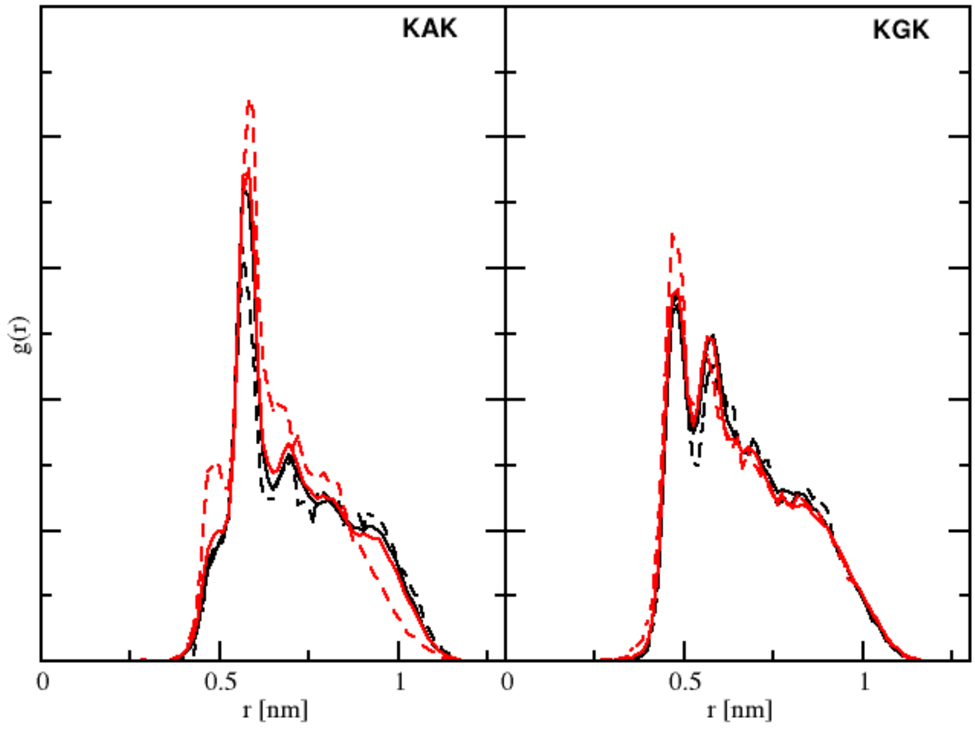

Supplement: Supplementary file 1 — Supporting Information [file JCC-37-2597-s001.pdf]
